# Subcutaneous batoclimab in generalized myasthenia gravis: Results from a Phase 2a trial with an open‐label extension

**DOI:** 10.1002/acn3.51946

**Published:** 2023-12-07

**Authors:** Richard J. Nowak, Ari Breiner, Vera Bril, Jeffrey A. Allen, Shaida Khan, Todd Levine, Daniel H. Jacobs, Gregory Sahagian, Zaeem A. Siddiqi, Jing Xu, William L. Macias, Michael Benatar, ASCEND MG Study Group, Laurence Adams, Angela Genge, Ali Habib, John L. Hinton, Tomas H. Holmlund, Daniel H. Jacobs, Dale J. Lange, Michael W. Nicolle, Han C. Phan, Nicholas J. Silvestri, George A. Small, Sharon Yegiaian

**Affiliations:** ^1^ Department of Neurology Yale University School of Medicine New Haven Connecticut USA; ^2^ Division of Neurology, Department of Medicine The Ottawa Hospital and Ottawa Research Institute, University of Ottawa Ottawa Ontario Canada; ^3^ Ellen & Martin Prosserman Centre for Neuromuscular Diseases University Health Network, University of Toronto Toronto Ontario Canada; ^4^ Department of Neurology University of Minnesota Minneapolis Minnesota USA; ^5^ Department of Neurology UT Southwestern Medical Center Dallas Texas USA; ^6^ HonorHealth Neurology dba Phoenix Neurological Associates Phoenix Arizona USA; ^7^ College of Medicine University of Central Florida Orlando Florida USA; ^8^ The Neurology Center of Southern California Carlsbad California USA; ^9^ Division of Neurology, Department of Medicine University of Alberta Hospital Edmonton Alberta Canada; ^10^ Immunovant Inc. New York New York USA; ^11^ Department of Neurology University of Miami Miami Florida USA

## Abstract

**Objectives:**

To assess the safety, tolerability, and key pharmacodynamic effects of subcutaneous batoclimab, a fully human anti‐neonatal Fc receptor monoclonal antibody, in patients with generalized myasthenia gravis and anti‐acetylcholine receptor antibodies.

**Methods:**

A Phase 2a, proof‐of‐concept, randomized, double‐blind, placebo‐controlled trial is described. Eligible patients were randomized (1:1:1) to receive once‐weekly subcutaneous injections of batoclimab 340 mg, batoclimab 680 mg, or matching placebo for 6 weeks. Subsequently, all patients could enter an open‐label extension study where they received batoclimab 340 mg once every 2 weeks for 6 weeks. Primary endpoints were safety, tolerability, and change from baseline in total immunoglobulin G, immunoglobulin G subclasses, and anti‐acetylcholine receptor antibodies at 6 weeks post‐baseline. Secondary endpoints included changes from baseline to 6 weeks post‐baseline for Myasthenia Gravis Activities of Daily Living, Quantitative Myasthenia Gravis, Myasthenia Gravis Composite, and revised 15‐item Myasthenia Gravis Quality of Life scores.

**Results:**

Seventeen patients were randomized to batoclimab 680 mg (*n* = 6), batoclimab 340 mg (*n* = 5), or placebo (*n* = 6). Batoclimab was associated with significantly greater reductions in total immunoglobulin G and anti‐acetylcholine receptor antibodies from baseline to 6 weeks post‐baseline than placebo. Reductions in immunoglobulin G subclasses were generally consistent with total immunoglobulin G. While clinical measures showed directionally favorable improvements over time, the study was not powered to draw conclusions about therapeutic efficacy. No safety issues were identified.

**Interpretation:**

The safety profile, pharmacodynamics, and preliminary clinical benefits observed in this study support further investigation of subcutaneous batoclimab injections as a potential patient‐administered therapy for seropositive generalized myasthenia gravis.

## Introduction

Myasthenia gravis (MG) is a chronic autoimmune neuromuscular disease that causes fatigable muscle weakness. Extraocular and eyelid muscles are particularly susceptible to weakness, though other muscles for chewing, talking, and swallowing are also frequently involved.^1^ The spectrum of symptoms ranges from a purely ocular form to generalized disease with severe weakness of the limb, bulbar, and respiratory muscles, and there is potential for significant morbidity and mortality.^1^, ^2^ Autoimmune MG results from the presence of autoantibodies recognizing proteins in the motor endplate at the neuromuscular junction, which leads to impaired neuromuscular transmission.^1^ Most commonly, in ≈80% of MG patients, anti‐acetylcholine receptor (AChR) antibodies (immunoglobulin G; IgG) are detected.^2^, ^3^, ^4^

The neonatal Fc receptor (FcRn) is critical to the regulation of IgG levels in the serum. FcRn regulates IgG degradation, thereby prolonging its circulating half‐life. Targeting the FcRn pathway dramatically reduces circulating IgG, thus supporting its evaluation for the treatment of autoantibody‐mediated autoimmune diseases.^5^ Batoclimab (RVT‐1401; Immunovant Inc., New York, NY, USA) is a fully human anti‐FcRn monoclonal antibody being developed as a low‐volume subcutaneous (SC) injection that functions by inhibiting the binding of IgG to FcRn. This results in the rapid catabolism of IgG via lysosomal degradation.^6^ In patients with MG, anti‐FcRn therapy is expected to lead to a significant reduction in the levels of various pathogenic IgG, including the anti‐AChR‐IgG and the anti‐muscle‐specific tyrosine kinase (MuSK)‐IgG, both of which have been identified as drivers of disease pathology in various subtypes of MG.^1^, ^2^ In a first‐in‐human study in healthy volunteers, once‐weekly SC injection of batoclimab, administered at doses of 340 mg or 680 mg, reduced total IgG concentrations by an average of 63% and 78%, respectively, at 3 weeks post‐baseline.^6^

The primary objectives of this study were to assess the safety and tolerability profile of low‐volume SC injections of batoclimab and to determine its effects on total IgG, IgG subclasses (1–4), and anti‐AChR‐IgG antibody levels in patients with MG. The secondary objectives were to assess the pharmacokinetics (PK) of SC batoclimab and to examine its effects on MG‐specific outcome measures to gather insights into the design of a possible efficacy trial in the future.

## Methods

### Study design

**Figure 1:**
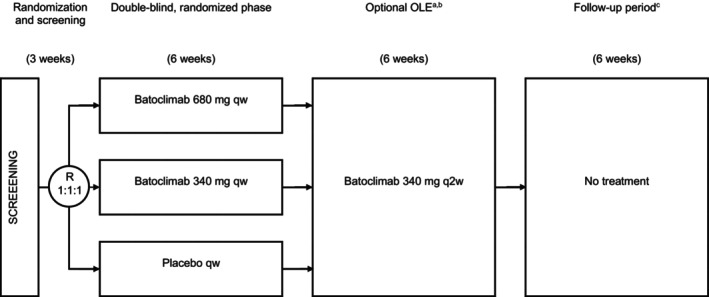
Schematic design and patient flow for the double‐blind Phase 2a trial with OLE. OLE, open‐label extension; q2w, once every 2 weeks; qw, once weekly; R, randomization. ^a^Patients not entering the OLE entered follow‐up; ^b^Patients not entering the OLE were planned to not receive study treatment; ^c^Planned follow‐up for patients not entering the OLE was 12 weeks.

This proof‐of‐concept, randomized, double‐blind, three‐arm, placebo‐controlled, Phase 2a trial was performed to evaluate the safety, tolerability, PK, pharmacodynamics (PD), and preliminary efficacy of batoclimab in patients with anti‐AChR antibody‐positive gMG (Clinicaltrials.gov: NCT03863080). In total, 17 patients from 11 centers in two countries (Canada and United States) were randomized 1:1:1 to receive once‐weekly SC injections of batoclimab 340 mg, batoclimab 680 mg, or matching placebo for 6 weeks. Subsequently, all patients had the option to enter a 6‐week open‐label extension (OLE), which was designed to assess the transition from a weekly regimen to an every‐other‐week dosing regimen. Patients entering the OLE received 3 more doses of batoclimab 340 mg once every 2 weeks for 6 weeks, followed by 6 weeks of treatment‐free follow‐up (Fig. 1). Patients who did not participate in the OLE entered a 12‐week treatment‐free follow‐up period.

### Standard protocol approvals, registrations, and patient consent

Independent ethics committees/institutional review boards provided written approval for the study protocol and all amendments. The study was also performed in compliance with the protocol, Good Clinical Practice, Declaration of Helsinki, International Council for Harmonisation, and other applicable regulatory requirements. Written informed consent was obtained from all patients prior to entering the study. The study was registered on 5 March 2019, the first patient was enrolled on 10 July 2019, and the last patient visit was on 21 December 2020.

### Key inclusion and exclusion criteria

Men and women aged ≥18 years were eligible for this study if they had Myasthenia Gravis Foundation of America (MGFA) Class II−IVa MG; had a Quantitative Myasthenia Gravis (QMG) score ≥ 12 at screening and baseline; were receiving stable doses of MG treatment before randomization; and were currently not in need of respiratory support (investigator assessment). Participants were also required to have a positive serologic test for anti‐AChR antibodies at screening, with at least one of the following: history of abnormal neuromuscular transmission testing (single‐fiber electromyography or repetitive nerve stimulation), history of positive edrophonium chloride testing, or demonstrated improvement in MG signs on oral cholinesterase inhibitors.

Key exclusion criteria included having received rituximab, belimumab, eculizumab, or any monoclonal antibody/Fc‐fusion biologic agent for immunomodulation within 6 months before first dose; tacrolimus within 1 week before the first dose or at any study visit during treatment; parenterally delivered immunoglobulins within 4 weeks before the screening; and having had a thymectomy <12 months before screening, or a splenectomy. Criteria are available at Clinicaltrials.gov (NCT03863080).

All patients who completed the double‐blind study were eligible to enter the OLE study period.

### Randomization and masking

Randomization within the double‐blind phase was performed using an interactive web response system with central randomization. Patients were randomly assigned (1:1:1) to receive batoclimab 680 mg/week (two syringes each containing 2 mL study drug), batoclimab 340 mg/week (one syringe containing 2 mL study drug and 1 syringe containing 2 mL matched placebo), or placebo alone (two syringes each containing 2 mL matched placebo). The same formulation was used for matched placebo as for batoclimab, but without the active ingredient. An unblinded medical monitor reviewed IgG, albumin, total protein, alkaline phosphatase, and anti‐AChR antibody data after screening on an ongoing basis for safety. However, to maintain blinding, investigators, study site personnel, and patients remained blinded to these data throughout both the double‐blind phase and OLE study.

### Procedures

The study included a screening period of 3 weeks to evaluate eligibility, a 6‐week double‐blind, placebo‐controlled treatment period, an OLE treatment period of 6 weeks, and a follow‐up period of 6 weeks (Fig. 1). Patients who chose not to enter the OLE study were followed for 12 weeks after their last dose of study treatment. During the 6‐week double‐blind treatment phase, eligible patients received 6 doses of batoclimab 680 mg, batoclimab 340 mg, or a matching placebo administered as a SC injection every week. Patients who entered the 6‐week OLE study received 3 additional doses of batoclimab 340 mg by SC injection every 2 weeks. Adverse events (AEs), serious AEs (SAEs), electrocardiograms, clinical laboratory assessments, and vital signs were evaluated at prespecified time points and summarized descriptively. Frequencies and proportions of patients with AEs were described for each treatment arm by preferred term and system organ class using the Medical Dictionary for Regulatory Authorities (MedDRA version 21.1), which was also used to summarize individual listings of all AEs and SAEs. The severity (mild [1], moderate [2], severe/medically significant [3], life‐threatening [4], death [5]) and causality (probably, possibly, not related) of every AE were assessed by the investigators.

Anti‐AChR antibody levels were quantitated from serum samples. Specific assays that were used were the Acetylcholine Receptor Autoantibody (AChRAb) RIA Assay Kit (KRONUS, Star, ID, USA) for AChR antibodies, and the Optilite® immunoturbidimetric assay for IgG subclasses (The Binding Site Group Ltd., Birmingham, United Kingdom), both at Quest Diagnostics, San Juan Capistrano, CA, USA. The immunoturbidimetric immunoglobulin assay (F. Hoffmann‐La Roche Ltd., Basel, Switzerland) at Eurofins Central Lab, Lancaster, PA, USA was used for total IgG quantification.

Serum compound concentration–time data were analyzed via noncompartmental methods using Phoenix WinNonlin version 8.1 (Certera, Princeton, NJ, USA). PK parameters for batoclimab assessed in the study included area under the serum concentration−time curve (AUC_(0−t)_), maximum observed plasma concentration (C_max_), time to reach C_max_ (t_max_), and plasma concentration before dosing (C_trough_).

PD assessments included serum IgG, IgG subclasses (1–4), and anti‐AChR concentrations, summarized as both raw values and percentage change from baseline (intra‐patient assessment). Clinical efficacy was assessed using the MG Activities of Daily Living (MG‐ADL) score (range 0–24),^7^, ^8^ the QMG score (range 0–39),^9^ and the Myasthenia Gravis Composite (MGC) disease severity score (range 0–50).^10^ Additionally, the revised 15‐item Myasthenia Gravis Quality of Life (MG‐QoL15r) scale (range 0–30),^11^ evaluating the effect of batoclimab on quality of life, and a brief survey on patient experience with the SC injections during the study, were utilized.

### Outcomes

Primary endpoints included safety and tolerability, and change from baseline in total IgG, IgG subclasses (1–4), and anti‐AChR antibodies at 6 weeks post‐baseline. Secondary endpoints included immunogenicity (determined by percentage change from baseline in anti‐batoclimab antibodies) and characterization of any anti‐batoclimab antibodies to confirm neutralization potential at 6 weeks post‐baseline. Additional secondary endpoints included PK/PD variables and change in scores from baseline to 6 weeks post‐baseline for QMG, MG‐ADL, MGC, and MG‐QoL15r.

### Statistical analysis

The sample size for this study was intended to generate descriptive data that would evaluate the proof‐of‐concept that SC injection of batoclimab can successfully reduce pathogenic antibodies in the population studied. The sample size of 14 patients receiving batoclimab and 7 patients receiving placebo was posited to allow the current study to show a 33% difference between either batoclimab arm and the placebo arm, assuming 90% power, equal standard deviations (SDs) of 20, and an alpha of 0.05 using a two‐sided z‐test for the primary endpoint of percentage change from baseline in IgG at 6 weeks post‐baseline.

As specified in the statistical analysis plan, the full analysis set included all randomized patients who received at least 1 dose of randomized study medication with at least one valid post‐treatment value. An interim analysis of all endpoints was performed after the last patient completed the double‐blind treatment period and constituted the primary analysis because of COVID‐19‐related disruptions. (See Impact of COVID‐19 pandemic below.) As this analysis occurred at the end of the double‐blind phase, no adjustments to the alpha level were necessary. A final analysis was performed after the completion of the study and database lock. Comparisons between active treatment arms and placebo for efficacy endpoints were performed using an analysis of covariance (ANCOVA) model with treatment and baseline values incorporated in the model; an alpha of 0.05 was used to determine statistical significance. Categorical values were analyzed using Fisher's exact test. Follow‐up and OLE, PK, and PD data were analyzed descriptively. All data were processed and summarized using SAS®, version 9.4.

### Impact of COVID‐19 pandemic

Contingency measures were implemented to conduct the study during the COVID‐19 pandemic. For patients in the OLE or follow‐up period of the study, virtual, telephone, or telehealth visits were allowed as an option for sites and patients in which COVID‐19 or travel restrictions prevented an in‐person visit. In addition, collecting blood samples at the patient's home or an alternative location by home health care workers or study personnel was allowed. While the above changes to the conduct of the study were allowed to minimize disruptions, implementation varied from site to site. Study sites were instructed to consult with their IRB/IEC, ensure compliance with local laws and regulations, and assess local capabilities prior to implementation. A total of 34 remote monitoring visits were completed and, among 6 patients, a total of 17 visits were not completed across the 3 study periods. If data were missing for 2 or more patients, missing data were predicted using multiple imputation methodology for the primary endpoint at 6 weeks post‐baseline.

Enrollment was closed early due to the COVID‐19 pandemic. In accordance with FDA guidelines on considerations for clinical trials during the COVID‐19 public health emergency, the sponsor decided to halt further recruitment and perform an analyses of 16 patients who had completed the double‐blind treatment period. (The target enrollment was 21 patients).

## Results

### Baseline characteristics

**Figure 2:**
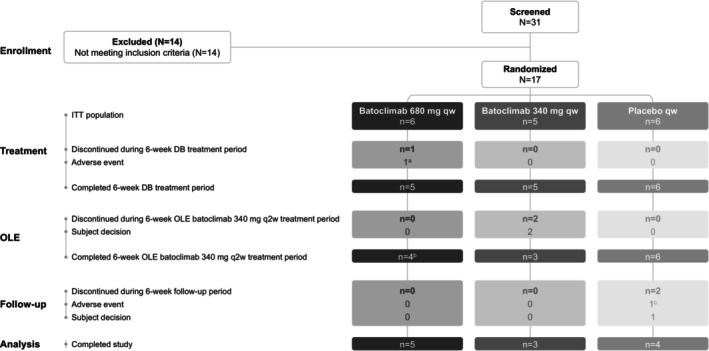
CONSORT diagram. DB, double‐blind; ITT, intention‐to‐treat; OLE, open‐label extension; q2w, once every 2 weeks; qw, once weekly. ^a^Herpes simplex; ^b^One patient who did not enter the OLE was included in the 12‐week treatment‐free follow‐up period, and completed the study; ^c^Worsening of myasthenia gravis.

Of 31 patients screened, 17 met the eligibility criteria and were randomized to receive double‐blind study treatment, and 12 patients completed all three study periods (Fig. 2). The mean (±SD) age at enrollment was 41.0 (±15.4) years in the placebo arm versus 64.5 (±18.8) in the combined batoclimab arms; 2 (33.3%) versus 8 (72.7%) were male; and 4 (66.7%) versus 7 (63.6%) were MGFA Class III, respectively. The mean (±SD) time since MG symptom onset was 7.5 (±5.6) years in the placebo arm versus 5.3 (±4.4) years in the combined batoclimab arms. All patients were white and, where reported, non‐Hispanic/Latino. Six patients had undergone thymectomy: 4 patients randomized to placebo, and 1 patient each randomized to the batoclimab 680 mg and batoclimab 340 mg arms. Most patients had prior/concomitant acetylcholinesterase inhibitor (15 out of 17 [88.2%]), prednisone (11 out of 17 [64.7%]), and nonsteroidal immunosuppressant (9 out of 17 [52.9%]) use. There were some differences between the treatment arms at baseline (Table 1). Notably, compared with the other arms, mean age and the proportion of men were higher in the batoclimab 680 mg arm, and the mean (SD) time since onset of MG was longer in the placebo arm compared with those in the combined batoclimab arm. Additionally, the batoclimab 340 mg arm had higher mean MGC and MG‐QOL15r scores at baseline than the other arms.

**Table 1:** Baseline characteristics (ITT population).

Parameter	Randomized arm for double‐blind phase^a^			
	Batoclimab 680 mg qw → 340 mg q2w *n* = 6	Batoclimab 340 mg qw → 340 mg q2w *n* = 5	Batoclimab combined *n* = 11	Placebo qw → 340 mg q2w *n* = 6
Mean (±SD) age, years	70.8 (±14.2)	56.8 (±22.2)	64.5 (±18.8)	41.0 (±15.4)
Male, *n* (%)	5 (83.3)	3 (60.0)	8 (72.7)	2 (33.3)
White, *n* (%)	6 (100.0)	5 (100.0)	11 (100.0)	6 (100.0)
Mean (±SD) time since MG onset, years	5.2 (±3.6)	5.4 (±5.6)	5.3 (±4.4)	7.5 (±5.6)
MGFA classification at screening				
II	2 (33.3)	1 (20.0)	3 (27.3)	2 (33.3)
III	3 (50.0)	4 (80.0)	7 (63.6)	4 (66.7)
IV	1 (16.7)	0 (0.0)	1 (9.1)	0 (0.0)
Symptom severity scales, Mean (±SD)				
QMG score	16.2 (±2.2)	16.6 (±3.4)	16.4 (±2.7)	17.2 (±3.2)
MG‐ADL score	7.2 (±2.4)	9.6 (±4.0)	8.3 (±3.3)	7.3 (±3.9)
MGC score	15.0 (±2.6)	19.6 (±5.0)	17.1 (±4.4)	16.8 (±5.3)
MG‐QOL15r score	13.5 (±8.5)	20.4 (±8.1)	16.6 (±8.7)	17.0 (±8.8)
Prior/concomitant MG treatments, *n* (%)				
Acetylcholinesterase inhibitors	5 (83.3)	5 (100.0)	10 (90.9)	5 (83.3)
Predisone	5 (83.3)	2 (40.0)	7 (63.6)	4 (66.7)
Nonsteroidal ISAs^b^	4 (66.6)	3 (60.0)	7 (63.7)	2 (33.3)

ISA, immunosuppressant agent; ITT, intention‐to‐treat; MG, myasthenia gravis; MG‐ADL, Myasthenia Gravis‐Activities of Daily Living; MG‐QOL15r, revised 15‐item Myasthenia Gravis Quality of Life; MGC, Myasthenia Gravis Composite; QMG, Quantitative Myasthenia Gravis; qw, once weekly; q2w, once every 2 weeks.

^a^ All patients completing the double‐blind phase were eligible to enter the OLE.

^b^ Includes azathioprine, mycophenolate mofetil, mycophenolic acid, mycophenolate sodium.

### Safety

Batoclimab was generally well tolerated throughout the study. The frequencies of patients experiencing any AEs and treatment‐related AEs (TRAEs) were well balanced between treatment arms, including the placebo arm, in the double‐blind phase (Table 2). Other than injection‐site reactions, there were no TRAEs reported during the OLE. Two patients discontinued due to AEs: 1 during the double‐blind phase and 1 during the follow‐up phase. A further 3 patients withdrew because of personal decisions, including 2 during the OLE and 1 during follow‐up.

**Table 2:** Safety profile.

Patients, *n* (%)	Double‐blind phase			OLE	Follow‐up
	Batoclimab 680 mg qw *n* = 6	Batoclimab 340 mg qw *n* = 5	Placebo qw *n* = 6	Batoclimab 340 mg q2w *n* = 15	No treatment *n* = 13
Any AE	5 (83.3)	4 (80.0)	5 (83.3)	7 (46.7)	6 (46.2)
Any TRAE	2 (33.3)	3 (60.0)	3 (50.0)	3 (20.0)	–
TRAEs by severity					
Grade 1–2	2 (33.3)	3 (60.0)	3 (50.0)	3 (20.0)	
Grade ≥3	0	0	0	0	
SAEs	1 (16.7)^a^	0 (0.0)	0 (0.0)	0	1 (7.7)^b^
AEs leading to treatment discontinuation	1 (16.7)^a^	0	0	0	0
TRAEs					
Headache	0	0	2 (33.3)	0	–
Constipation	0	0	1 (16.7)	0	
Decreased appetite	1 (16.7)	0	0	0	
Decreased complement activity	0	0	1 (16.7)	0	
Diarrhea	0	0	1 (16.7)	0	
Increased RBC count	0	1 (20.0)	0	0	
Muscle spasms	0	1 (20.0)	0	0	
Urinary tract infection	0	1 (20.0)	0	0	
Viral upper respiratory tract infection	1 (16.7)	0	0	0	
Pyuria	0	1 (20.0)	0	0	
Treatment‐related injection‐site reactions					
Any	1 (16.7)	2 (40.0)	1 (16.7)	3 (20.0)	–
Erythema	1 (16.7)	2 (40.0)	1 (16.7)	2 (13.3)	
Swelling	0	1 (20.0)	0	1 (6.7)	
Bruising	0	1 (20.0)	0	0	
Pain	0	0	1 (16.7)	0	

AE, adverse event; OLE, open‐label extension; q2w, once every 2 weeks; qw, once weekly; RBC, red blood cell; TRAE, treatment‐related AE.

^a^ *Herpes simplex* infection possibly related to therapy.

^b^ Grade 3 Worsening of MG in a patient who received placebo in the double‐blind period and batoclimab 340 mg q2w in the OLE. The patient discontinued early from the OLE follow‐up (Week 16). The event was considered unrelated to therapy.

One patient experienced a serious AE of *herpes simplex* infection during the double‐blind phase (batoclimab 680 mg arm), which was discovered incidentally during an ophthalmic examination while being hospitalized for acute generalized weakness and recurrent falls. This patient had numerous comorbidities (uncontrolled diabetes, hypertension, atrial fibrillation, Stage 3 chronic kidney disease, dyslipidemia, peripheral neuropathy, osteoporosis) and was taking several concomitant medications (prednisone, mycophenolate mofetil, pyridostigmine, apixaban, irbesartan, amlodipine). The event of *herpes simplex* was treated with a 14‐day course of acyclovir and erythromycin ointment in the left eye, during which time the event resolved, and the patient was subsequently discharged from the hospital; study treatment was withdrawn (Fig. 2; Table 2). This event was considered possibly treatment‐related by the investigator.

During the follow‐up phase, a serious AE (worsening of MG) leading to early study discontinuation was reported in a patient who received placebo during the double‐blind phase and batoclimab 340 mg every 2 weeks during the OLE; the patient was hospitalized, and the event resolved. This event was considered unrelated to study drug.

Reductions in serum albumin were observed during the study, with maximum mean reductions from baseline observed at Week 4 in the batoclimab 680‐mg group (mean [±SD] concentration, 31.6 [±3.1] g/L; mean [SD] change from baseline, −12.0 [±2.6] g/L) and at Week 5 in the 340‐mg group (mean [±SD] concentration, 35.2 [±1.3] g/L; mean [SD] change from baseline, −11.2 [±3.4] g/L). Low albumin concentrations (defined as <32 g/L) were observed in 4 out of 11 patients receiving batoclimab (all in the 680‐mg group) during the double‐blind phase, including 2 patients with nadirs of 27 g/L. At the end of the follow‐up period (i.e., 6 weeks after the last dose of batoclimab), mean albumin concentrations returned back toward baseline levels. No patients had an AE related to hypoalbuminemia.

The impact of batoclimab treatment on lipids was examined in a post hoc analysis using a limited number of available samples. Results indicated a dose‐dependent increase in total cholesterol, LDL‐C, and HDL‐C, with greater increases observed in patients receiving batoclimab 680 mg. In all instances, the lipid values in patients who had received batoclimab in the double‐blind treatment period returned to baseline values by the end of the follow‐up period at Week 17. While interpretation of these results is limited, given that data were only available for 1 or 2 patients at many timepoints, the findings are consistent with other studies of batoclimab.^12^

### Pharmacodynamics: Change in IgG and anti‐AChR antibody levels

**Figure 3:**
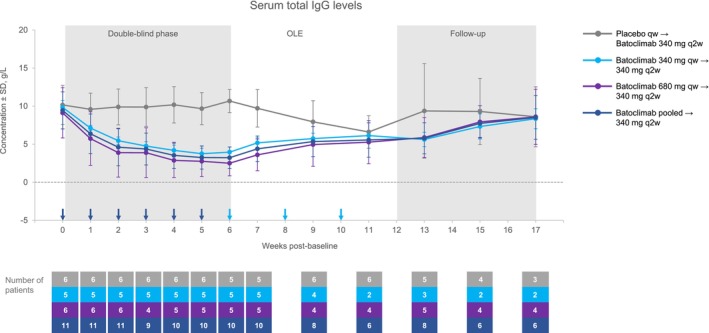
Serum total IgG levels over time. IgG, immunoglobulin G; OLE, open‐label extension; q2w, once every 2 weeks; qw, once weekly; SD, standard deviation.

In the double‐blind phase, administration of batoclimab at both doses was associated with decreases in serum concentrations of total IgG that were significantly different (*p* < 0.0001) when compared with the placebo arm at 6 weeks post‐baseline (Fig. 3). The least squares (LS) mean estimate of the percentage changes from baseline to 6 weeks post‐baseline were −70.0% (95% confidence interval [CI]: −76.7%, −63.2%) for batoclimab (combined arm) and −3.2% (95% CI: −9.0%, 2.7%) for placebo. During the OLE, patients switching from placebo to batoclimab experienced reductions in total IgG when initiated on batoclimab 340 mg every 2 weeks. Total IgG concentrations in patients originally randomized to batoclimab trended up during the follow‐up period after discontinuation of treatment but had not returned to baseline levels by the end of the follow‐up period (17 weeks post‐baseline).

**Figure 4:**
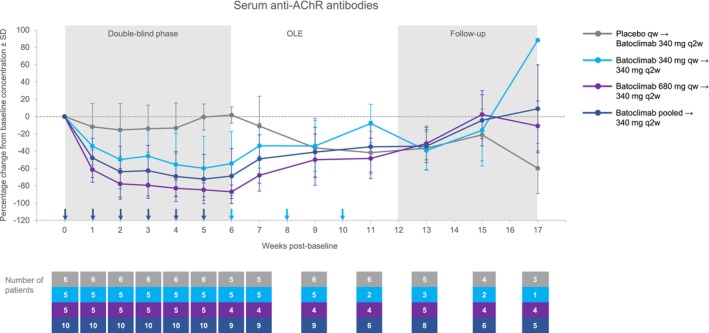
Serum anti‐AChR antibodies over time. AChR, acetylcholine receptor; OLE, open‐label extension; q2w, once every 2 weeks; qw, once weekly; SD, standard deviation.

Reductions in IgG subclasses (1–4) with batoclimab were generally consistent with total IgG during the double‐blind phase. Percentage changes from baseline to 6 weeks post‐baseline (mean [±SD]) for the combined batoclimab arm versus the placebo arm were as follows: IgG1, −71.5% (±9.9%) versus −2.1% (±8.7%); IgG2, −61.0% (±13.7%) versus −2.3% (±3.2%); IgG3, −76.1% (±11.5%) versus 1.2% (±14.4%); and IgG4, −55.6% (±16.8%) versus −4.9% (±9.8%). Batoclimab administration had no observable effects on serum IgA and IgM concentrations (data not shown). Serum anti‐AChR antibody concentrations also declined significantly in the batoclimab arms (340 mg: *p* = 0.0009; 680 mg: *p* < 0.0001) relative to placebo during the double‐blind phase at 6 weeks post‐baseline (Fig. 4). The LS mean estimate of the percentage change from baseline for the combined batoclimab arm was −68.3% (95% CI: −88.0%, −48.5%), and 15.9% (95% CI: −8.4%, 40.3%) for the placebo arm at 6 weeks post‐baseline. In patients who received placebo during the double‐blind phase and were switched to active treatment with batoclimab 340 mg every 2 weeks during the OLE, anti‐AChR antibody concentrations decreased by 35.3% (SD: 17.51%) at the end of the OLE, resulting in similar concentrations among the 3 arms by the end of this phase. Upon treatment discontinuation in the follow‐up period, antibody concentrations increased in patients who received batoclimab 340 mg during the double‐blind period.

### Clinical outcomes

**Figure 5:**
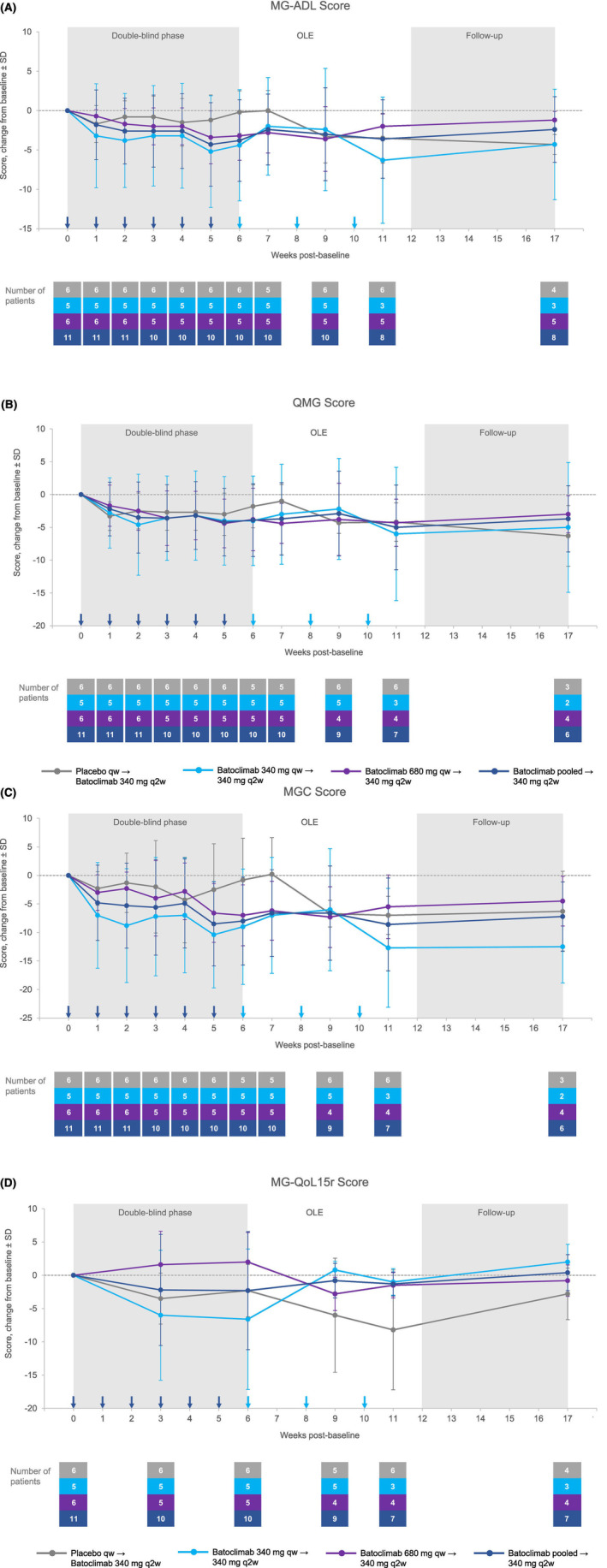
Clinical outcome measures over time. (A) MG‐ADL score; (B) QMG score; (C) MGC score; (D) MG‐QoL15r. MG, myasthenia gravis; MG‐ADL, MG Activities of Daily Living; MGC, Myasthenia Gravis Composite; MG‐QoL 15r, revised 15‐item Myasthenia Gravis Quality of Life; OLE, open‐label extension; q2w, once every 2 weeks; QMG, Quantitative Myasthenia Gravis; qw, once weekly; SD, standard deviation.

MG‐specific clinical outcome measures (MG‐ADL, QMG, and MGC) showed that patients who received batoclimab had lower scores versus patients who received placebo during the double‐blind phase, although differences (change in mean [±SD]) were not statistically significant at the end of the double‐blind phase (6 weeks post‐baseline) (Fig. 5A–C): MG‐ADL score, −3.8 (±5.2) versus −0.2 (±3.0); QMG score, −3.9 (±5.6) versus −1.8 (±3.3); and MGC score, −8.0 (±7.7) versus −0.8 (±7.3), for the combined batoclimab and placebo arms, respectively. There was no observable effect on the patient‐assessed quality of life measure in the batoclimab arms versus placebo (Fig. 5D).

### Classification of evidence

The primary research question was to examine the safety and tolerability and PD effects of batoclimab in patients with generalized MG. This study provides Class I evidence that, for patients with generalized MG, SC batoclimab injections are well tolerated, and that IgG and anti‐AChR antibody levels were significantly reduced at 6 weeks post‐baseline.

## Discussion

This proof‐of‐concept study in patients with generalized MG demonstrated that a low‐volume SC injection of batoclimab was well tolerated and associated with significant reductions in both total IgG and anti‐AChR antibody concentrations. The safety profile of batoclimab was generally favorable and similar to the placebo arm throughout the study. There was one serious AE of herpes simplex infection, which led to hospitalization and discontinuation of study treatment and was considered by the investigator to be possibly related to treatment. Reductions in serum albumin were also observed, which is an expected effect based on the MOA of batoclimab, as FcRn recycles albumin using the same mechanism as for IgG. Low albumin concentrations were observed in 4 patients that received batoclimab 680 mg; however, albumin concentrations returned to baseline levels by end of the follow‐up period. No albumin‐related AEs were reported, and no low albumin concentrations were observed at the 340 mg batoclimab dose.

Dose‐related elevations in serum lipids have been observed with batoclimab treatment, including in the Phase 2b trial in thyroid eye disease, which led to the early termination of that trial.^12^ However, post hoc analyses of lipids data found that those changes were dose‐related and reversible after stopping treatment. Consistent with those results, post hoc lipid analyses using a limited number of available samples from this study also suggested dose‐dependent increases in total cholesterol, LDL‐C, and HDL‐C. These observations are likely related to reductions in serum albumin,^13^, ^14^ and were reversible upon discontinuation of the drug. Moreover, preliminary results from a study in healthy volunteers have shown that lipid increases with batoclimab treatment may be prevented by co‐administration of atorvastatin.^15^

Batoclimab treatment led to rapid declines in total IgG and anti‐AChR antibody concentrations, which were evident within 2 weeks after the first dose. These effects versus placebo were sustained throughout the double‐blind phase. Patients randomized to placebo received batoclimab during the OLE and also demonstrated antibody reductions when initiated on the lower dose of 340 mg every 2 weeks. The differential effects of batoclimab on individual subclasses of IgG were generally consistent with total IgG. As expected, batoclimab had no effect on IgA and IgM concentrations. The population that received batoclimab 680 mg was older and had a greater proportion of men compared with the batoclimab 340 mg and placebo groups; this is likely a product of randomization in a small study and does not affect safety and efficacy endpoints given that similar trends were observed in the batoclimab 340 mg group.

FcRn inhibition has been shown to be an effective mechanism by which to treat MG.^16^, ^17^ Initial FcRn inhibitors were developed to be administered as infusions.^16^, ^17^, ^18^, ^19^, ^20^, ^21^ To further advance the application of anti‐FcRn therapy, batoclimab was designed to be administrated as a low‐volume SC injection, which has the potential for home self‐administration, allowing greater convenience for the patient and lower healthcare resource utilization. Additionally, this may allow for individualized treatment regimens with different dosing (680 or 340 mg once weekly, and 340 mg every 2 weeks). In the field of immunology, real‐world evidence has shown that injection of biologic agents, as opposed to IV or SC infusion, may save both provider and facility time by reducing drug preparation and administration time, subsequently lowering costs for formulations delivered via small‐volume injection relative to formulations of the same agent requiring infusion. Moreover, patients have reported better quality of life and expressed a preference for SC injection over IV as a route of administration.^22^, ^23^ A proven and available SC injection treatment option remains an unmet need at the present time.

The study has several limitations. The sample size was small and there were some imbalances across groups with respect to baseline characteristics. In particular, patients who received placebo during the double‐blind period were younger than those who received batoclimab. In addition, the mean (SD) time since MG onset was approximately 5 years in batoclimab patients and approximately 7 years in placebo patients. The heterogeneity of the patient population and short duration of the treatment period may also help to explain the smaller effect on clinical measures that were observed with the 680‐mg versus the 340‐mg dose of batoclimab, despite greater reductions in IgG and anti‐AChR antibodies with the 680‐mg dose. As this Phase 2a proof‐of‐concept study was designed primarily as a PK/PD and safety trial, it was not powered to evaluate clinical efficacy. Additionally, there was a lack of racial and ethnic diversity that limits the generalizability of the results. That said, based on the findings of this study, further evaluation of batoclimab as a possible future treatment option for patients with gMG is being explored in a registrational Phase 3 trial (Clinicaltrials.gov: NCT05403541).

In conclusion, the results of this Phase 2a trial demonstrated that SC batoclimab is well tolerated and significantly reduced IgG and anti‐AChR antibody levels in patients with gMG, supporting its further investigation as a potential patient‐administered therapeutic agent for MG.

## Author Contributions

Richard J. Nowak: Drafting/revision of the manuscript; major role in the acquisition of data; conceptualization of study design; analysis/interpretation of data. Ari Breiner: Drafting/revision of the manuscript; major role in the acquisition of data; analysis/interpretation of data. Vera Bril: Drafting/revision of the manuscript; major role in the acquisition of data; analysis/interpretation of data. Jeffrey A. Allen: Drafting/revision of the manuscript for content; major role in the acquisition of data; analysis/interpretation of data. Shaida Khan: Drafting/revision of the manuscript; major role in the acquisition of data; analysis/interpretation of data. Todd Levine: Drafting/revision of the manuscript; major role in the acquisition of data; analysis/interpretation of data. Daniel H. Jacobs: Drafting/revision of the manuscript; major role in the acquisition of data; analysis/interpretation of data. Gregory Sahagian: Drafting/revision of the manuscript; major role in the acquisition of data; analysis/interpretation of data. Zaeem A. Siddiqi: Drafting/revision of the manuscript; major role in the acquisition of data; analysis/interpretation of data. Jing Xu: Drafting/revision of the manuscript; analysis/interpretation of data. William L. Macias: Drafting/revision of the manuscript; analysis/interpretation of data. Michael Benatar: Drafting/revision of the manuscript; conceptualization of study design; analysis/interpretation of data.

## Conflict of Interest Statement

Richard J. Nowak reports research support from the National Institutes of Health, Genentech, Inc., Alexion Pharmaceuticals, Inc., argenx, Annexon Biosciences, Inc., Ra Pharmaceuticals, Inc. (now UCB S.A.), the Myasthenia Gravis Foundation of America, Inc., Momenta Pharmaceuticals, Inc., Immunovant, Inc., Grifols, S.A., and Viela Bio, Inc. (Horizon Therapeutics plc). Dr. Nowak has also served as a consultant and advisor for Alexion Pharmaceuticals, Inc., argenx, Cabaletta Bio, Inc., CSL Behring, Grifols, S.A., Ra Pharmaceuticals, Inc. (now UCB S.A.), Immunovant, Inc., Momenta Pharmaceuticals, Inc., and Viela Bio, Inc. (Horizon Therapeutics plc). Ari Breiner reports grant support from the Muscular Dystrophy Association and Muscular Dystrophy Canada. Dr. Breiner has served as an advisor for Alnylam, and has received speaker fees from Mitsubishi‐Tanabe. Vera Bril has served as a consultant for Grifols, CSL, UCB, argenx, Takeda, Alnylam Octapharma, Pfizer, Powell Mansfield Inc, Akcea, Ionis Immunovant, Sanofi, Momenta (J&J), Roche, Janssen, AZ‐Alexion, and NovoNordisk. Dr. Bril also reports research support from AZ‐Alexion, Grifols, CSL, UCB, argenx, Takeda, Octapharma, Akcea, Momenta (J&J), Immunovant, and Ionis. Jeffrey A. Allen has served as a consultant for argenx, Alexion, Annexon, CSL Behring, Takeda, ImmunoPharma, Grifols, Pfizer, Johnson and Johnson. Shaida Khan has served as a consultant for UCB Pharmaceuticals. Dr. Khan has served on advisory boards for Alexion, UCB Pharmaceuticals, argenx, and Immunovant, Inc. Dr. Khan has received research support from the Fichtenbaum Charitable Trust. Todd Levine has served as a consultant for Immunovant, Inc. Daniel H. Jacobs has no disclosures to report for the last 3 years. Gregory Sahagian received consulting and/or research fees from Biogen, UCB, argenx, Alexion and Immunovant. Zaeem A. Siddiqi has served as a consultant for Pfizer, Grifols, CSL Behring, UCB, Takeda, Alnylam. Octapharma, and AZ‐Alexion and has received research support from Pfizer AZ‐Alexion, Grifols, CSL Behring, UCB, Takeda, and Octapharma. Jing Xu: Employee of Immunovant, Inc (New York, NY). William L. Macias: Employee of Immunovant, Inc (New York, NY). Michael Benatar reports grants from the National Institutes of Health and the Muscular Dystrophy Association; as well as consulting fees for Alector, Alexion, Annexon, Arrowhead, Biogen, Cartesian, Denali, Eli Lilly, Horizon, Immunovant, Janssen, Novartis, Orphazyme, Roche, Sanofi, Takeda, UCB and UniQure. The University of Miami has licensed intellectual property to Biogen to support design of the ATLAS study.
